# The D-SPECT SH reconstruction protocol: improved quantification of small left ventricle volumes

**DOI:** 10.1186/s40658-023-00606-y

**Published:** 2024-01-08

**Authors:** Yan Huang, Han Zhang, Xueping Hu, Shanshan Qin, Fan Hu, Yuchen Li, Haidong Cai, Kuangyu Shi, Fei Yu

**Affiliations:** 1 Medical College, Anhui University of Science and Technology, Huainan, China; 2 Department of Nuclear Medicine, Shanghai Tenth People’s Hospital, Tongji University School of Medicine, Shanghai, China; 3 Institute of Nuclear Medicine, Tongji University School of Medicine, Yanchang RD.301, Shanghai, 200072 China; 4 Department of Informatics, Technical University of Munich, Munich, Germany; 5 Department of Nuclear Medicine, University of Bern, Bern, Switzerland

**Keywords:** Gate myocardial perfusion single-photon computed tomography, Small left ventricle volume, Reconstruction algorithm, Left ventricle ejection fraction

## Abstract

**Background:**

Due to spatial resolution limitations, conventional NaI-SPECT typically overestimates the left ventricular (LV) ejection fraction (EF) in patients with small LV volumes. The purpose of this study was to explore the clinical application value of the small heart (SH) reconstruction protocol embedded in the postprocessing procedure of D-SPECT.

**Methods:**

We retrospectively analyzed patients who undergo both D-SPECT and echocardiography (Echo) within one week. Patients with small LV volume were defined as those with a rest end-systolic volume (rESV) ≤ 25 mL and underwent reconstruction using the standard (SD) reconstruction protocol. The SH protocol was deemed successful in correcting the LVEF value if it decreased by 5% or more compared to the SD protocol. The ROC curve was used to calculate the optimal cutoff value of the SH protocol. LVEF, ESV and EDV were computed with SD and SH, respectively. Echo was performed as a reference, and Echo-LVEF, ESV, and EDV were calculated using the Teichholz formula. One-way ANOVA was used to compare these parameters among the three groups.

**Results:**

The final study included 209 patients (73.21% female, age 67.34 ± 7.85 years). Compared with the SD protocol, the SH protocol significantly decreased LVEF (67.43 ± 7.38% vs. 71.30 ± 7.61%, *p* < 0.001). The optimal cutoff value for using the SH protocol was rESV > 17 mL (AUC = 0.651, sensitivity = 78.43%, specificity = 45.57%, *p* = 0.001). In the subgroup of rESV > 17 mL, there was no significant difference in LVEF (61.84 ± 4.67% vs. 62.83 ± 2.85%, *p* = 0.481) between the SH protocol and Echo, and no significant difference was observed in rESV (26.92 ± 3.25 mL vs. 27.94 ± 7.96 mL, *p* = 0.60) between the SH protocol and Echo.

**Conclusion:**

This pilot study demonstrated that the SH reconstruction protocol was able to effectively correct the overestimation of LVEF in patients with small LV volumes. Particularly, in the rESV > 17 mL subgroup, the time and computing power waste could be reduced while still ensuring the accuracy of the LVEF value and image quality.

## Background

Validation studies have been conducted to demonstrate the effectiveness of gated myocardial perfusion single-photon computed tomography (GSPECT) myocardial perfusion imaging (MPI) in assessing and classifying the risk of patients with suspected or known coronary artery disease (CAD) [1–4]. It has been established that functional parameters, such as left ventricle (LV) ejection fraction (EF) and LV volumes, are in agreement with those from echocardiography (Echo), cardiac magnetic resonance imaging (CMR) and left ventriculography [5–7]. However, an issue with GSPECT is that it tends to underestimate LV end-systolic volume (ESV) and end-diastolic volume (EDV) and overestimate LVEF in patients with small LV volumes when using the standard (SD) reconstruction protocol, thus leading to a misdiagnosis and affecting the prognostic evaluation of these patients, referred to as the “small heart effect” [8–10].

Numerous researchers have endeavored to tackle this issue from the perspectives of refining scanning and reconstruction techniques [11–13]. The small heart (SH) reconstruction protocol, developed by Spectrum Dynamics Medical Company, has improved the spatial resolution of reconstructed images when compared to the SD reconstruction protocol, making it possible to accurately delineate the endocardium in patients with small LV volumes. To date, no studies have been conducted to investigate the SH reconstruction protocol’s usage in clinical application for patients with small LV volumes.

To help fill this gap, the aim of this study was to validate the feasibility of the SH reconstruction protocol to enhance the quantitative accuracy of the LVEF and LV volumes in patients with small LV volumes. Additionally, we further explored the application value of the SH reconstruction protocol in certain patients.

## Materials and methods

### Patient population

**Fig. 1:**
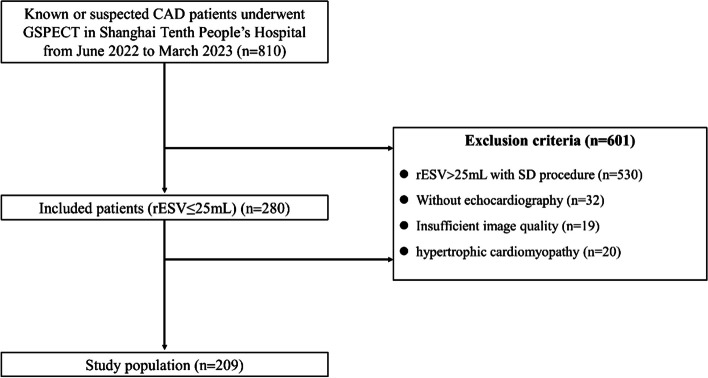
Patient flowchart

We performed a retrospective study of 810 consecutive patients with suspected or known CAD who were referred for rest/stress ^99m^Tc-sestamibi (MIBI) GSPECT from June 2022 to March 2023 at the Department of Nuclear Medicine, Shanghai Tenth People’s Hospital. Patients were excluded if rest end-systolic volume (rESV) > 25 mL was reconstructed using the SD protocol (*n* = 530), if echocardiography was not performed (*n* = 32), if imaging quality was insufficient (inferior wall and ventricular septal artifacts were too heavy to accurately delineate the endocardium; GSPECT quantification failed due to severe arrhythmia) (*n* = 19), or if hypertrophic cardiomyopathy was present (*n* = 20). LVs with an rESV ≤ 25 mL as calculated using the SD reconstruction protocol were defined as small. After applying the exclusion criteria, a total of 209 small heart patients were eligible for inclusion in our study (Fig. 1). The ethics committee of Shanghai Tenth People’s Hospital approved the protocol under the approval number SHSY-IEC-4.1/21-126/01.

### Imaging acquisition

Equipment: The D-SPECT Cardiac System Model 003 (Spectrum Dynamics Medical Ltd., Israel, Caesarea, Serial Number: 5217) was equipped with 9 mobile blocks of pixelated CZT detectors (pixel size, 2.46 mm × 2.46 mm). Each collimated detector column rotates and translates independently (up to 110°), allowing for the observation of objects of interest from hundreds of different angles [14].

Radiotracer drug: ^99m^Tc-sestamibi (MIBI) (Shanghai Xinke Pharmaceutical Co., Ltd., Shanghai, China).

Stress test drug: Adenosine injection (Penglai Nuokang Pharmaceutical Co., Ltd., Penglai, Shandong Province, China).

Scanning Protocol: All patients that were included were following a one-day rest/stress GSPECT MPI protocol, and the rest images were used in this study. Before examination, patients refrained from theophylline and caffeine-containing beverages for at least 12 h and fasted for at least 3 h [15]. An experienced nuclear medicine technologist conducted the rest GSPECT MPI acquisition, which began 60–90 min after an intravenous injection of 370 MBq of ^99m^Tc-MIBI. The stress protocol was started approximately 60 min after the injection of adenosine and radiopharmaceutical. To minimize the impact of the liver and intestine on the heart readings, the patient was instructed to consume a fatty diet 30 min before the examination. The technologist was blinded to all other imaging data and the clinical status of the patient [16]. The energy window was set to 140 keV ± 10%, the size of the acquisition matrix was 64 × 64, and the cardiac cycle was divided into 8 frames for ECG gating. The scan time was set to the time until a total of 1.5 M counts were reached for the LV. No attenuation or scatter corrections were applied [17].

### Reconstruction parameters

After the images were acquired, the data was transmitted to the Spectrum Dynamics Medical Processing Station and reconstructed with SD and SH reconstruction protocols. The SD is performed using 32 subsets and 4 iterations. The SH is performed using 32 subsets and iterated 9 times. Inter-iteration filtering is applied by convolution with a smoothing kernel with a central value of (1 − *ω*), where *ω* is 0.125 [17, 18]. The only distinction between the two reconstruction protocols is the number of iterations. Both of them are based on the maximum-likelihood expectation maximization (MLEM) algorithm [19]. Both the SD and SH reconstruction protocols have a fixed number of subsets and iterations, with the reconstructed voxel size being 4.92 × 4.92 × 4.92 mm. Automatic processing was initially used for the software, but when the wall tracing was visually judged to be inappropriate, the operator modified the ventricular border surrounding the ventricle and reprocessed the edge. Then ventricular systolic function parameters, LVEF; ventricular volume parameters, rest EDV, and rESV were acquired automatically by subsequent processing in DICOM format using the quantitative perfusion SPECT software (QGS, Cedars-Sinai Medical Center). If rESV ≤ 25 mL was calculated by SD, the SH reconstruction protocol was chosen.

### Echocardiography

M-mode and two-dimensional echocardiograms were used to measure LV function parameters. The M-shaped sampling line was perpendicular to the left ventricular long axis, placed at the level of the chordae tendineae. Dd (end-diastolic diameter) and Ds (end-systolic diameter). EDV and ESV were measured using the Teichholz formula: EDV = 7.0 × Dd^3^/(2.4 + Dd), ESV = 7.0 × Ds^3^/(2.4 + Ds). LVEF = (EDV-ESV)/EDV*100% [20].

### Statistical analysis

After verifying the normal distribution with the Kolmogorov − Smirnov test, continuous variables were presented as the mean ± standard deviation and were compared using the independent samples* t* test for two groups. Cardiac function parameters of Echo were used as the reference standard, and one-way ANOVA was used for the analysis of the three groups, while the Bonferroni correction was used for post hoc comparison. A ΔLVEF change > 5% between the SD and SH reconstruction protocols was considered to be corrected successfully. Receiver operator characteristic (ROC) analysis [21] was used to derive the optimal cutoff point of rESV based on the two-category data (1 represented ΔLVEF > 5%; 0 represented ΔLVEF ≤ 5%). A *p* value of < 0.05 was considered statistically significant. All statistical analyzes were performed using SPSS 24.0 for Windows (SPSS Inc., Chicago, IL, United States) and GraphPad Prism 8.0.1.

## Results

### Patient characteristics

This study included 209 patients with small LV volumes (mean age 67.34 ± 7.85 years, 73.21% female), 130 (62.20%) of whom had hypertension, 42 (20.10%) of whom had diabetes mellitus and 5 (2.39%) of whom had dyslipidemia. The patients’ baseline characteristics are summarized in Table 1.

**Table 1:** Clinical characteristics of the study population

Patient characteristics	*n* = 209
Age (mean ± SD)	67.34 ± 7.85
Female (%)	153(73.21)
Height (m)	1.62 ± 0.07
Weight (kg)	62.97 ± 10.45
BMI (kg/m^2^)	23.91 ± 3.33
BSA (m^2^)	1.64 ± 0.16
Hypertension (%)	130(62.20)
Diabetes mellitus (%)	42(20.10)
Dyslipidemia (%)	5 (2.39)
Family history of CAD	12(5.74)
Previous PCI	46(22.01)

Body surface area (BSA) = 0.0061 × Height (m) + 0.0128 × Weight (kg) − 0.1529. CAD: coronary artery disease

### Determination of parameters using SD, SH and echo in the overall population

**Fig. 2:**
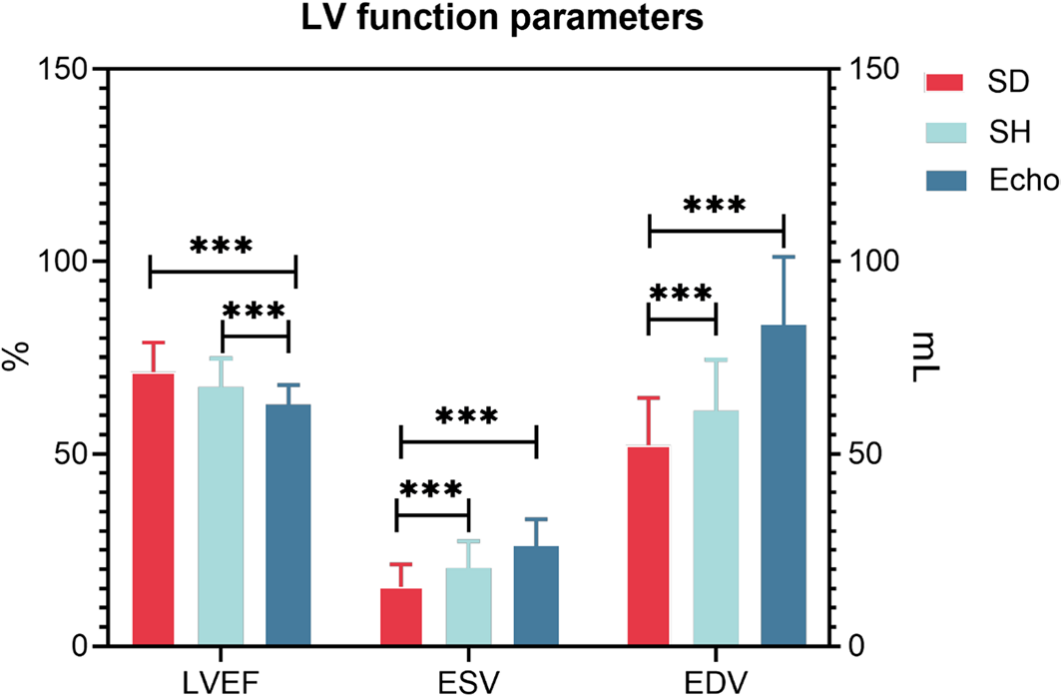
LV function parameters measured by SD, SH protocol and Echo in the overall population. *** represents *p* < 0.001

**Fig. 3:**
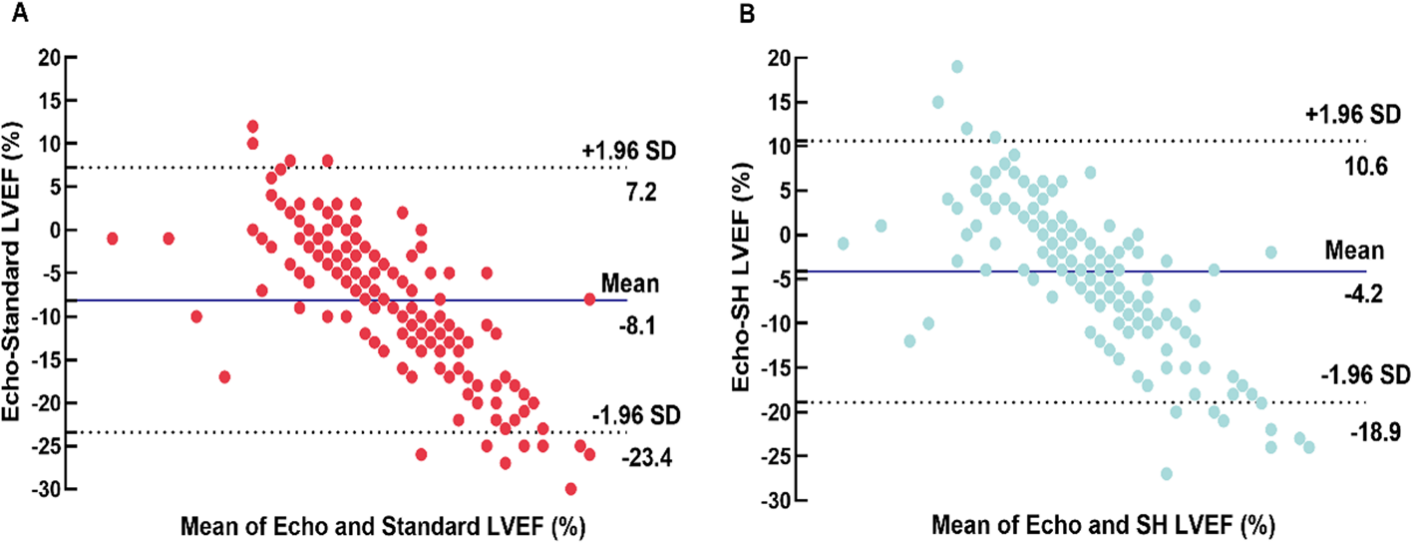
**A** Agreement for LVEF measurement: Bland-Atman plots for Echo and SD reconstruction protocol. **B** Bland-Atman plots for Echo and SH reconstruction protocol

**Fig. 4:**
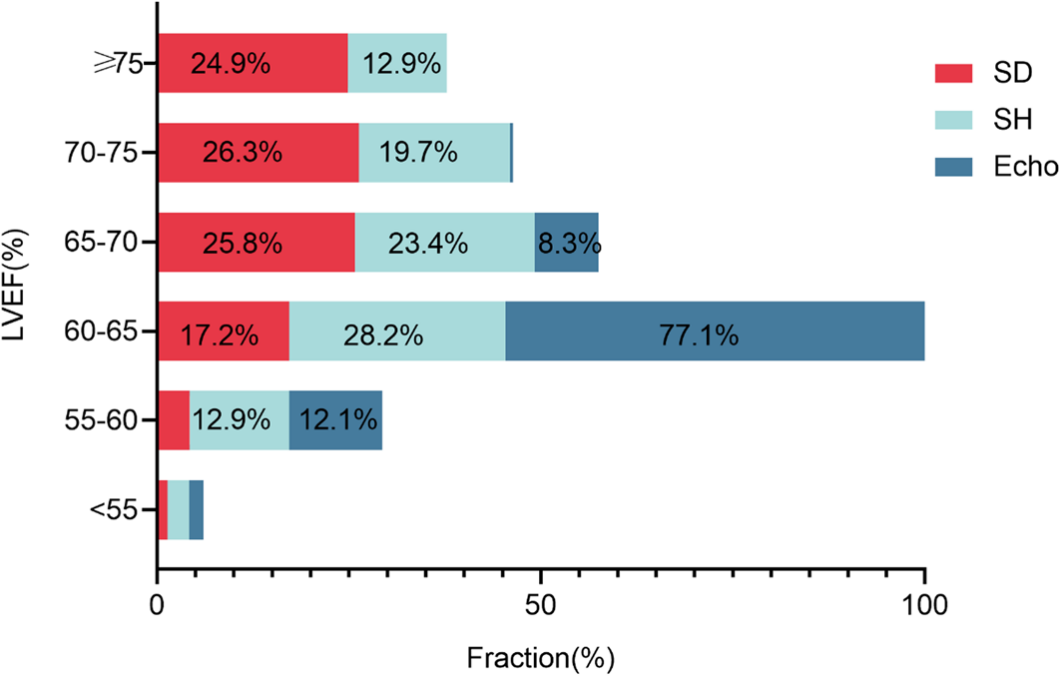
The LVEF was divided into 6 ranges, and the proportion of each range was used among the SD, SH protocol and Echo. The x-axis shows the percentage of patients, and the y-axis shows LVEF%

The LVEF was overestimated (71.30 ± 7.61% vs. 63.15 ± 2.65%, *p* < 0.001), and the ESV and EDV (15.45 ± 5.83 mL vs. 26.51 ± 6.04 mL, *p* < 0.001, 52.35 ± 12.25 mL vs. 84.98 ± 13.61 mL, *p* < 0.001) were underestimated by the SD reconstruction protocol when compared with Echo. The SH protocol partially mitigated the overestimation of LVEF caused by the “small heart effect”, and 24% of patients were successfully corrected. The mean SH-LVEF was 67.43 ± 7.38% in all patients with more minimal bias (-4.2%) and narrow BA-LA (-18.9% to 10.6%) compared with SD (Table 2, Figs. 2 and 3). Furthermore, the majority of LVEFs that were reconstructed using the SD protocol were within the 70–75% range, accounting for 26.32%. The majority of LVEFs that were reconstructed using the SH protocol were within the 60–65% range, accounting for 28.23%. The majority of LVEF measured by Echo was also within the 60–65% range, accounting for 77.07% (Fig. 4).

**Table 2:** Paired comparisons of the parameters obtained from SD, SH and Echo

Parameter	SD	SH	SD vs.SH	Echo	SH vs. Echo
LVEF (%)	71.30 ± 7.61	67.43 ± 7.38	*p* < 0.001	63.15 ± 2.65	*p* < 0.001
ESV (mL)	15.45 ± 5.83	20.32 ± 6.97	*p* < 0.001	26.51 ± 6.04	*p* < 0.001
EDV (mL)	52.35 ± 12.25	61.21 ± 13.27	*p* < 0.001	84.98 ± 13.61	*p* < 0.001

### The cutoff value of rESV following the SH reconstruction protocol

**Fig. 5:**
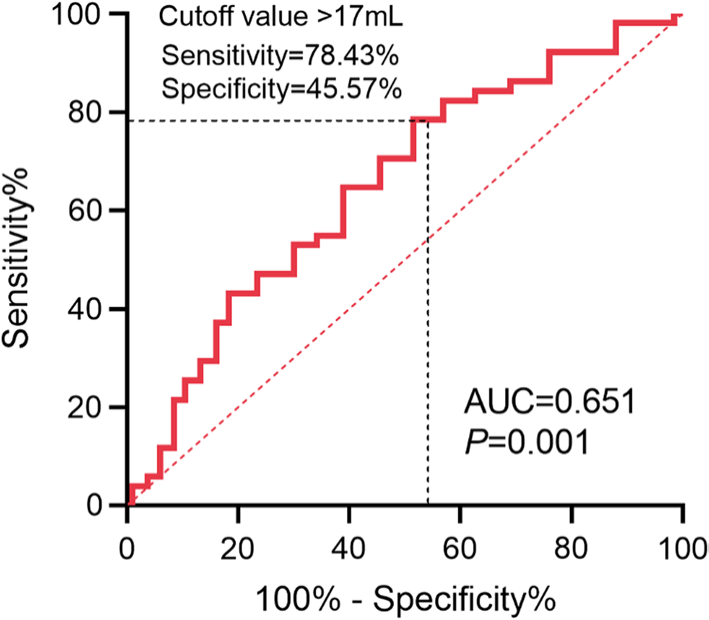
The optimized cutoff value of rESV reconstructed following the SH protocol

**Fig. 6:**
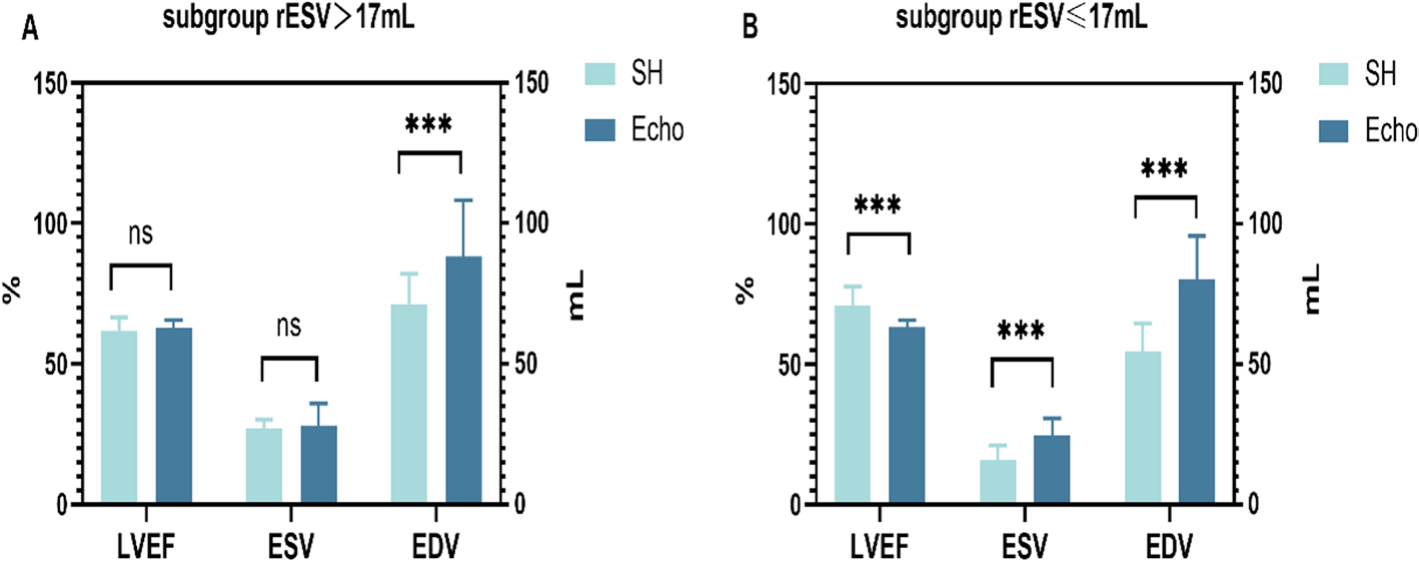
**A** In the rESV > 17 mL subgroup, the LV function parameters obtained with the SH protocol and Echo. **B** In the rESV ≤ 17 mL subgroup, the LV function parameters obtained using the two methods. *** represents *P* ≤ 0.001

The optimal cutoff value for rESV was 17 mL, with a sensitivity and specificity of 78.43% and 45.57%, respectively (Fig. 5). The rESV > 17 mL subgroup had 83 patients (39.71%), while the rESV ≤ 17 mL subgroup had 126 (60.29%). When comparing the SH protocol and Echo, no significant difference in LVEF and ESV was observed in the rESV > 17 mL subgroup (61.84 ± 4.67% vs. 62.83 ± 2.85%, *p* = 0.481; 26.92 ± 3.25 mL vs. 27.94 ± 7.96 mL, *p* = 0.596). The SH-EDV was slightly lower than the Echo-EDV (71.43 ± 10.66 mL vs. 88.24 ± 19.94 mL, *p* < 0.001). Nevertheless, the SD-LVEF was significantly higher than the Echo-LVEF (65.10 ± 4.67% vs. 62.83 ± 2.85%, *p* = 0.001), and the SD-ESV and SD-EDV were significantly lower than the Echo-ESV and Echo-EDV (21.27 ± 2.15 mL vs. 27.94 ± 7.96 mL, *p* < 0.001; 62.05 ± 9.81 vs. 88.24 ± 19.94, *p* < 0.001). In the rESV ≤ 17 mL subgroup, the SH-LVEF and SD-LVEF were higher than the Echo-LVEF (71.18 ± 6.52%, 75.37 ± 6.46% vs. 63.36 ± 2.50%, *p* < 0.001). Both the ESV (15.98 ± 5.09 mL, 11.62 ± 4.05 mL vs. 24.72 ± 5.99 mL, *p* < 0.001) and EDV (54.48 ± 10.16 mL, 45.96 ± 9.10 mL vs. 80.13 ± 15.54 mL, *p* < 0.001) were underestimated. These results are shown in Table 3 and Fig. 6.

**Table 3:** Parameters calculated by SD, SH and Echo respectively in both subgroups. The bold showed that for the subgroup with rESV >17mL, the LVEF and ESV values were not significantly different between the SH reconstruction protocol and Echo, whereas the SD reconstruction protocol and Echo had a statistically significant difference

Parameter	Group	SD	SH	SD vs.SH	Echo	SH vs. Echo
	All	71.30 ± 7.61	67.43 ± 7.38	*p* < 0.001	63.15 ± 2.65	*p* < 0.001
LVEF (%)	**rESV > 17**	**65.10 ± 4.67**	**61.84 ± 4.67**	*p* =** 0.001**	**62.83 ± 2.85**	*p* =** 0.48**
	rESV ≤ 17	75.37 ± 6.46	71.18 ± 6.52	*p* < 0.001	63.36 ± 2.50	*p* < 0.001
	All	15.45 ± 5.83	20.32 ± 6.97	*p* < 0.001	26.00 ± 7.00	*p* < 0.001
ESV (mL)	**rESV > 17**	**21.27 ± 2.15**	**26.92 ± 3.25**	*p* <** 0.001**	**27.94 ± 7.96**	*p* =** 0.60**
	rESV ≤ 17	11.62 ± 4.05	15.98 ± 5.09	*p* < 0.001	24.72 ± 5.99	*p* < 0.001
	All	52.35 ± 12.25	61.21 ± 13.27	*p* < 0.001	83.35 ± 17.83	*p* < 0.001
EDV (mL)	rESV > 17	62.05 ± 9.81	71.43 ± 10.66	*p* < 0.001	88.24 ± 19.94	*p* < 0.001
	rESV ≤ 17	45.96 ± 9.10	54.48 ± 10.16	*p* < 0.001	80.13 ± 15.54	*p* < 0.001

**Fig. 7:**
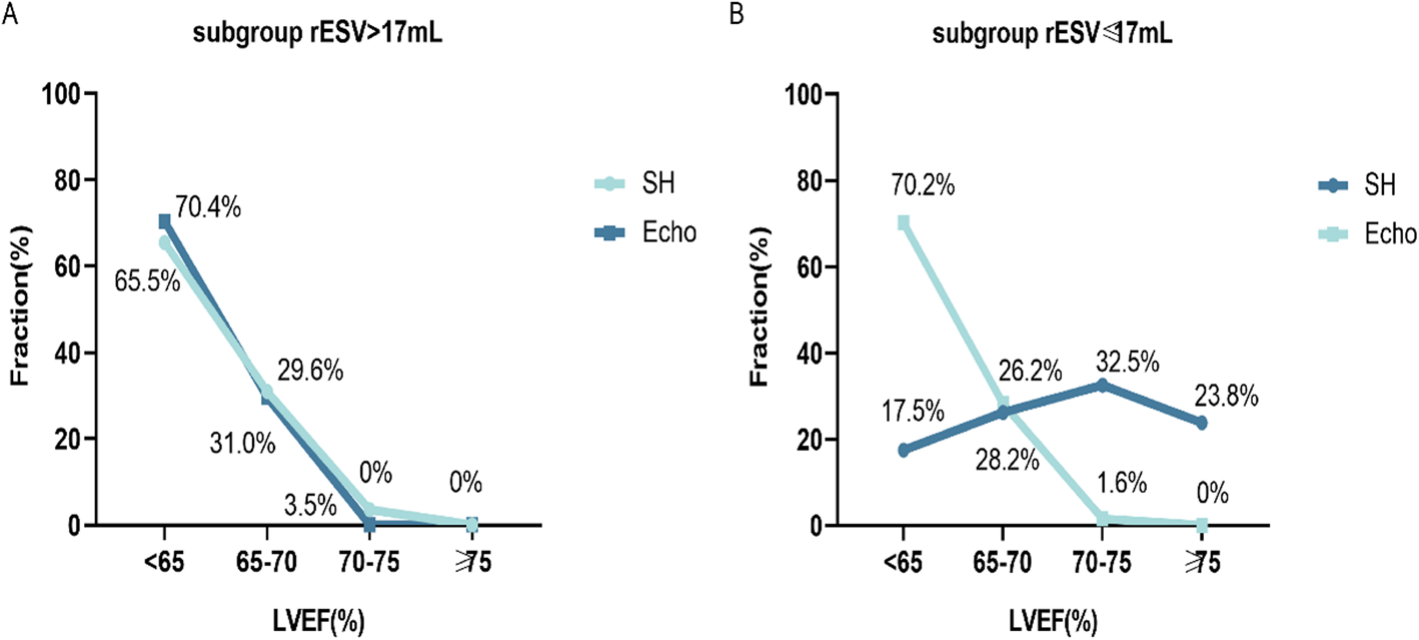
The distribution of LVEF values over four intervals calculated using the SH protocol and Echo. **A** In the rESV > 17 mL subgroup, the SH protocol and Echo were used to measure a consistent distribution of LVEF across three ranges. **B** In the rESV ≤ 17 mL subgroup, the SH protocol and Echo were used to measure an inconsistent distribution of LVEF across three ranges. The “*Fraction*” on the y-axis was defined as the proportion of the number of patients in the total study population

**Fig. 8:**
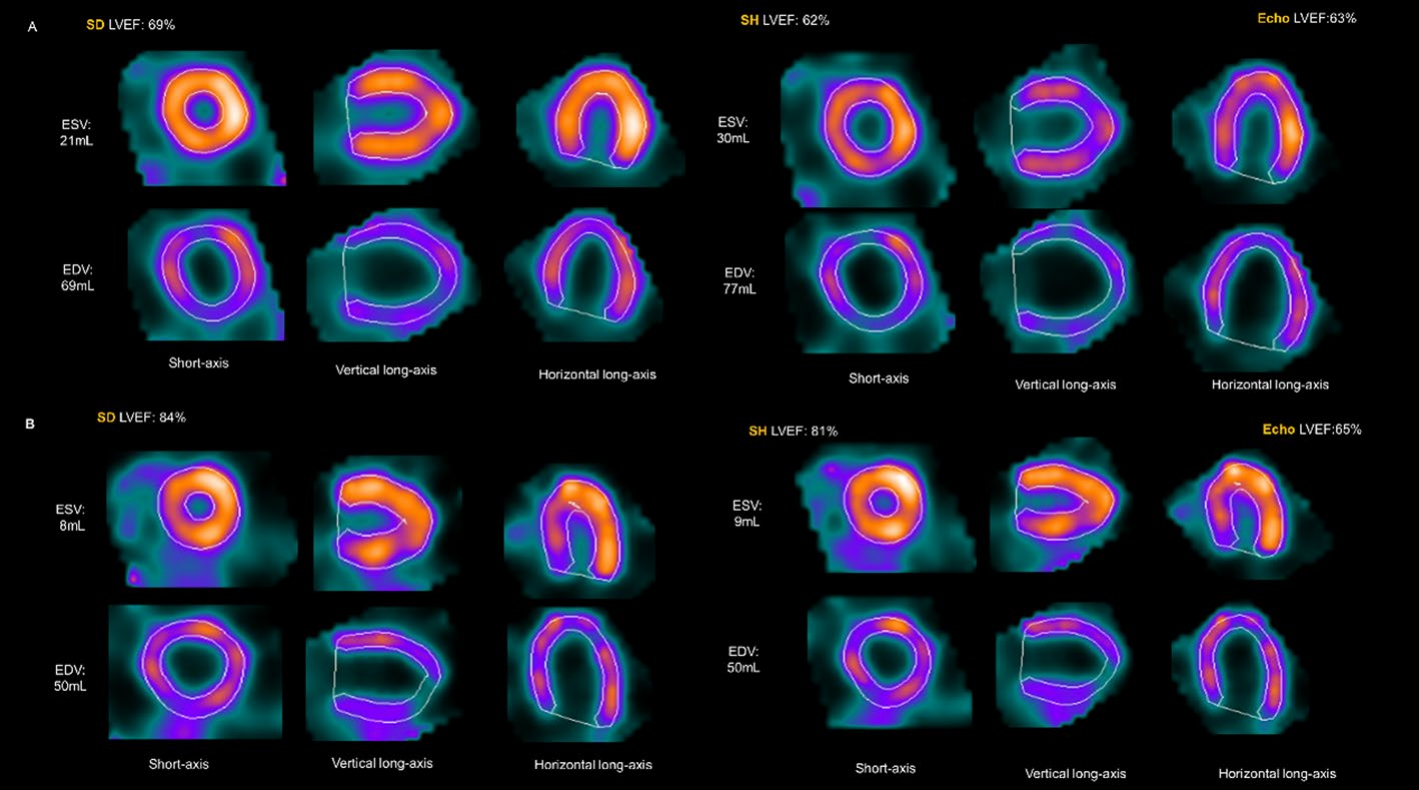
**A** A 61-year-old male patient. ESV = 21 mL calculated by the SD protocol, and the LVEFs calculated using the SD and SH protocols were 69% and 62%, respectively. Echo showed an EDV of 97 mL, an ESV of 35 mL, and a LVEF of 63% by using Teichholz’s formula. **B** A 63-year-old female patient. ESV = 8 mL calculated using the SD protocol, and the LVEFs calculated using the SD and SH protocols were 84% and 81%, respectively. Echo showed an EDV of 70 mL, an ESV of 22 mL, and a LVEF of 65%

The SH-LVEF distributions and Echo-LVEF distributions were further compared and analyzed in four ranges (< 65%, 65–70%, 70–75%, ≥ 75%), and there was no significant difference in the distribution of the rESV > 17 mL subgroup (*p* = 0.619). Nevertheless, a significant difference was observed in the rESV ≤ 17 mL subgroup (*p* < 0.001) (Fig. 7). Two case examples were used as supplementary explanations (Fig. 8). Figure A featured a 61-year-old male patient with LVEF, ESV and EDV results of 69%, of 21 mL and of 69 mL for the SD protocol, 62%, 30 mL, and 77 mL for the SH protocol and 63%, 35 mL, and 97 mL for the Echo, respectively. Figure B shows a 63-year-old female patient with LVEF, ESV and EDV results of LVEF 84%, ESV 8 mL, EDV 50 mL for the SD protocol, 81%, 9 mL and 50 mL for the SH protocol, and 65%, 22 mL and 70 mL for the echo, respectively.

## Discussion

Our study revealed three main points; first, the SH reconstruction protocol could reduce the overestimation of LVEF in patients with small LV volumes; second, the best threshold for using the SH reconstruction protocol is when rESV > 17 mL; third, after reconstruction following the SH protocol in the rESV > 17 mL subgroup, the LVEF and ESV were not significantly different from the gold-standards by Echo, although it underestimated the EDV.

### Small heart effect

LVEF typically plays a pivotal role in the diagnosis and prediction of cardiovascular disease, and incorrectly assessing LVEF may lead to faulty clinical decisions [22]. Although conventional NaI quantitative GSPECT has been shown to contain adequate diagnostic and prognostic information [23], limitations due to a number of physical factors, including photon scattering, insufficient spatial resolution, and partial volume effects [24, 25], can underestimate ESV in patients with a small LV volume, which consequently leads to frequent overestimation of LVEF. This condition is particularly common in Asian populations due to their generally smaller body size. Similar to the distribution of our study population (73% women), Nakajima et al. observed small LV volumes in 74% of Japanese women and 13% of men [25]. Kakhki et al. also found that in the Iranian population, where the likelihood of CAD was low, 85.4% had an ESV ≤ 25 mL (91% of women and 9% of men). Meanwhile, due to systematic and reconstruction spatial resolution limitations, the LV endocardial rim is blended together, thus making the ventricular cavity almost virtual, especially at end-systole [8].

In this regard, our study confirms that D-SPECT equipped with a new semiconductor CZT detector can solve the above problems to a certain extent. Not only designed to compensate for the lack of spatial resolution, the built-into SH reconstruction protocol has higher endocardial edge detection accuracy at end-systole, which allows for better correction of LVEF and mitigating the "small heart effect" and has enhanced clinical application in Asian populations.

### Comparison of LVEF detected by CZT-SPECT with that by other imaging methods

Many studies have compared the efficacy between CZT-SPECT using the SD reconstruction protocol and other imaging methods to evaluate LV volumes. Coupez et al. compared the D-SPECT-derived resting LVEF with Echo in patients with myocardial infarction (MI), and the results showed that D-SPECT with the SD reconstruction protocol tended to overestimate LVEF compared with Echo (55.6% vs. 51.3%), but there was no statistically significant difference (*p* = 0.34) [6]. Cochet et al. reported that Discovery NM 530C can accurately quantify LVEF in patients with known or suspected CAD but still underestimate LV volumes compared with CMR [26]. Claudin et al. also reported that there were significant underestimations of EDV (*p* < 0.001, mean difference with CMR: -46 ± 25 mL) and ESV (*p* < 0.001, mean difference with CMR: -20 ± 19 mL) using D-SPECT with the SD reconstruction protocol; the underestimations were markedly increased for smaller LV volumes, and the volume dependence was primarily for ESV (*p* = 0.04) rather than EDV (*p* = 0.1) in patients with known or suspected CAD [27]. Similar to previous studies, we found that the LV volumes measured by D-SPECT with the SD reconstruction protocol were underestimated compared with Echo (EDV 52.35 ± 12.25 mL vs. 83.35 ± 17.83 mL, *p* < 0.001; ESV 15.45 ± 5.83 mL vs. 26.00 ± 7.00 mL, *p* < 0.01), and the SH reconstruction protocol mainly corrected ESV (26.92 ± 3.25 mL vs. 27.94 ± 7.96 mL, *p* = 0.596). Consequently, in clinical practice, it is essential to select the right reconstruction protocol, and the SH reconstruction protocol has been found to be effective in patients with small LV volumes.

### Reconstructed spatial resolution

Reconstruction parameters are the central techniques for accurate assessment of LV functional parameters. The reconstructed spatial resolution was improved with more iterations. Compared to the SD protocol, the SH reconstruction protocol more than doubles the number of iterations (from 4 to 9). This incremental gain in the number of iterations results in sharper images, expands the measurement of the endocardial edge, and improves the accuracy of ESV and LVEF, bringing them closer to the true values. Wang et al. also reported that the full width at half-maximum (FWHM) decreased with an increasing number of iterations, and a better reconstructed spatial resolution could be obtained when the number of iterations was greater than 4 [28]. However, there is currently no unified standard for the optimal iteration number. Duarte et al. reported the use of 2 iterations with 10 or 12 subsets as the best match of the iterative algorithm when measuring the LVEF and LV volumes during GSPECT using a dynamic heart phantom [29]. A study from Bitarafan et al. reported similar results (2 iterations and 12 subsets) when compared with Echo [30]. Ceriani et al. reported that the combination of 10 iterations and 8 subsets produced the most accurate LVEF results using a dynamic heart phantom as the gold reference [31].

Nevertheless, our study also demonstrated that not all patients with small LV volumes benefit from the SH protocol, and the increase in the number of iterations does not seem to have a significant effect on the rESV ≤ 17 mL subgroups. The reason for this result is partly because the spatial resolution of D-SPECT is 5 mm, which is not very satisfactory for the outlining of particularly small cardiac chambers [32]. At the same time, the number of iterations and subsets of the SH protocol that have been developed thus far are not necessarily optimal solutions and need to be further explored in future clinical practice.

### Study limitations

This study has several limitations. First, due to its retrospective design, only Echo rather than CMR could be used as a criterion for the successful correction of LVEF, which may have a small impact on the findings. Second, due to the single center nature of this study, we opted for a single postprocessing software package (QGS). Previous research has revealed that D-SPECT with different programs has a degree of variability. Consequently, a large population and multicenter external validation is needed to validate the thresholds of rESV when using the SH reconstruction protocol. Third, the photon scattering problem has not yet been resolved in this paper, and deep learning techniques could be incorporated into the reconstruction program to improve it in the future.

## Conclusions

The performance of the SH reconstruction protocol was compared in this study with that of the SD reconstruction protocol in patients with small LV volumes. The findings indicate that the SH reconstruction protocol that was embedded in the D-SPECT postprocessing procedure can significantly improve the problem of insufficient myocardial delineation, especially for those with an rESV > 17 mL, allowing clinicians to make more accurate clinical decisions when evaluating patients with small LV volumes.

## Data Availability

The datasets generated during and/or analyzed during the current study are available from the corresponding author on reasonable request.

## Abbreviations

CAD: Coronary artery disease
GSPECT: Gated myocardial perfusion single-photon computed tomography
FWHM: Full width at half-maximum
LVEF: Left ventricle ejection fraction
ESV: End-systolic volume
EDV: End-diastolic volume
Echocardiography: Echo
